# CircRBM33 competitively binds miR-15a-5p to mediate EZH1 expression to ameliorate sepsis-induced acute lung injury

**DOI:** 10.1016/j.clinsp.2024.100550

**Published:** 2024-12-11

**Authors:** Jinquan Lin, Qiongying Wei, Zhipeng Fang

**Affiliations:** a Department of Trauma Center and Emergency Surgery, National Regional Medical Center, Binhai Campus of the First Affiliated Hospital, Fujian Medical University, Fuzhou City, Fujian Province, PR China; b Department of Pulmonary and Critical Care Medicine, Fujian Medical University Union Hospital, Fuzhou City, Fujian Province, PR China; c Department of Geriatrics, Fujian Medical University Union Hospital, Fuzhou City, Fujian Province, PR China; d Department of Trauma Center and Emergency Surgery, The First Affiliated Hospital, Fujian Medical University, Fuzhou City, Fujian Province, PR China

**Keywords:** CircRBM33, miR-15a-5p, EZH1, Septic acute lung injury, Inflammatory response, Oxidative stress

## Abstract

**Background:**

The study was to investigate circRBM33 in septic acute lung injury (ALI).

**Methods:**

Treatment of Murine Lung Epithelial-12 cells (MLE-12) cells was performed using 10 ng/mL Lipopolysaccharide (LPS). circRBM33, miR-15a-5p, and Enhancer of zeste homolog 1 (EZH1) were ascertained through RT-qPCR or Western blot analysis. The viability of MLE-12 cells was measured using the MTT assay, and their rate of apoptosis was ascertained through flow cytometry. B-cell lymphoma-2 (Bcl-2), and Bcl-2-associated X (Bax) were determined using Western blot analysis. Oxidative stress levels were assessed with ELISA kits, and levels of malondialdehyde(MDA) content, Superoxide Dismutase (SOD) activity, and glutathione (GSH) were detected. Dual luciferase reporter gene and RIP assays verified the targeting link between miR-15a-5p and circRBM33 or EZH1. The role of circRBM33 in ALI *in vivo* was determined by performing cecum ligation-perforation (CLP) surgery. HE staining, W/D pulmonary edema, and histological damage scores were taken to assess the extent of lung tissue damage. ELISA was performed to determine proinflammatory factors in lung tissue and cells.

**Results:**

CircRBM33 downregulation ameliorated ALI-induced edema, apoptotic, and inflammatory reactions in mouse lung tissues. In addition, apoptosis and inflammation mediated by LPS in MLE-12 cells were ameliorated by circRBM33 downregulation, whereas miR-15a-5p knockdown or EZH1 elevation eliminated the action of silencing circRBM33. circRBM33 mediated EZH1 expression by competitive adsorption of miR-15a-5p.

**Conclusion:**

CircRBM33 improves ALI in septic mice by targeting the miR-15a-5p/EZH1 axis.

## Introduction

Sepsis is an organ dysfunction caused by the host's malfunctioning response to infection. Sepsis may result in syndromes affecting multiple organs such as the lungs, kidneys, liver, and heart.^1^ Acute lung injury (ALI), marked by severe lung inflammation, presents a grave complication in sepsis. Around half of the sepsis patients experience ALI. Even with advancements in clinical treatment approaches, ALI continues to be a significant hurdle, exhibiting a mortality rate between 35% and 40%.^2^ The pursuit of potent therapeutic biomarkers is crucial for enhancing the disease's outlook. Reports indicate that ALI caused by sepsis is linked to the apoptosis of alveolar epithelial cells, unusual lung inflammation, and irregular apoptosis.^3^ ALI linked to sepsis is marked by an uncontrolled inflammatory reaction, with inflammatory agents believed to be crucial in starting, intensifying, and sustaining ALI caused by sepsis. Continuously high pro-inflammatory cytokines in the blood are known to significantly forecast mortality in individuals with ALI.^4^ Furthermore, suppressing nuclear factor-κB (NF-κB) activity could lessen lung inflammation and ALI caused by Lipopolysaccharide (LPS).^5^ Additionally, research indicated that apoptosis-related proteins, such as B-cell lymphoma-2 (Bcl-2) and Bcl-2-associated X (Bax) lead to widespread apoptosis and damage to the epithelium in alveolar epithelial cells affected by ALI.^6^ Additionally, research indicates that during sepsis, macrophages orchestrate the inflammatory reaction and play a role in intense inflammation and immunosuppression.^7^ Concurrently, the activation of LPS triggers a range of intracellular functions in macrophages. Consequently, the administration of LPS is extensively utilized in simulating lung damage linked to sepsis.

CircRNAs show high expression levels in eukaryotes, are well-preserved, and manifest in particular cells or during distinct developmental phases.^8^ CircRNAs serve a biological role as miRNA sponges, templates for translation, and regulators of gene transcription, engaging with proteins that bind to RNA. The characteristics of circRNAs render them ideal candidates for innovative diagnostic and treatment medications. circRNAs could potentially act as biomarkers for sepsis, controlling genes throughout the progression of ALI.^9^ As an illustration, circC3P1 diminishes inflammatory activities and cell death in septic ALI through the modulation of miR-21.^10^ In a mouse model for ALI sepsis, there is a notable increase in the expression of mmu-circ0001679.^11^ Furthermore, the research by Yuan et al.^12^ presents solid proof for identifying novel biomarkers and treatment targets in sepsis and septic shock. Through the examination of lung circRNA expression patterns in a rat model with CLP-induced lung injury, it was shown that circRBM33 had a high expression level in CLP mice, as evidenced by extensive sequencing. The bioinformatics examination of the circRBM33 source gene revealed a strong correlation between circRBM33 and the inflammatory response. Nonetheless, the role of circRBM33 in septic ALI remains a mystery.

MiRNAs, being non-coding, single-stranded endogenous RNAs, play a significant role in the molecular control mechanisms of diseases.^13^ miR-15a-5p is crucial in numerous diseases, including sepsis.^14^ Inflammation may serve as an indicative biomarker for sepsis, with numerous research indicating miRNAs play a crucial role in controlling the inflammatory reaction by inhibiting NF-κB activation,^15^ offering valuable insights for diagnosing and treating sepsis. Elevated levels of miR-15a-5p in cases of ALI caused by traumatic hemorrhagic shock or macrophage damage due to LPS indicate its significant role in septic ALI,^16^ serving as a key regulator.

The Polycomb (PcG) family member Enhancer of zeste homolog 1 (EZH1) plays a crucial role in gene silencing.^17^ As one of the catalytic subunits of Polycomb repressive complex 2 (PRC2), EZH1 represses transcription of target genes by triggering methylation of lysine residue 27 of histone H3. EZH1 is related to cancer diseases. Recent studies have illustrated that several oncogenes miR-200c^18^ and miR-26a^19^ have been identified as direct targets of EZH1.

The aim of this study was to explore the interactions between circRBM33 and miR-15a-5p, EZH1, and how circRBM33 promotes septic ALI via the miR-15a-5p/EZH1 axis.

## Materials and methods

### Sample collection

Sixty sepsis patients (34 males and 26 females) and 30 healthy controls (14 males and 16 females) were recruited for this study at the Binhai Campus of the First Affiliated Hospital, Fujian Medical University. All patients with sepsis were caused by bacterial infection and were excluded if they had other serious clinical diseases and had started any treatment within the past 3 months. Healthy controls demonstrated normal physiologic functioning on systemic physiologic examination. The study was approved by the Binhai Campus of the First Affiliated Hospital, Fujian Medical University Ethics Committee. All participants signed an informed consent form. Blood (3 ml) was withdrawn from patients and controls under fasting conditions on days 1 and 8 after admission (after 1 week of treatment). Plasma was separated by centrifuging blood samples with 1300 g for 15 min after mixing citric acid in a 10:1 ratio. All clinical studies followed the CONSORT guidelines.

### Cell treatment

Murine Lung Epithelial-12 cells (MLE-12; ATCC; VA, USA) were cultured in DMEM containing 10% FBS (Gibco), 100 mg/mL streptomycin, and 100 U/mL penicillin; Gibco, CA, USA) in culture (37 °C, 5% CO_2_). To model sepsis-induced ALI *in vitro*, MLE-12 cells underwent a 48-hour treatment with 10 ng/mL of LPS.

### Cell transfection

Small interfering oligonucleotides specifically targeting circRBM33 (si-circRBM33), miR-15a-5p mimic/inhibitor, EZH1 overexpression plasmid (pcDNA3.1-EZH1), and negative controls were provided by GenePharma (Shanghai, China). Transfection was carried out at 37 °C using Lipofectamine 3000 (Invitrogen, USA). RT-qPCR and Western blot were performed to assess the transfection efficiency after 24 h

### RT-qPCR

Using Trizol reagent (Thermo, USA), total RNA was isolated from lung tissues and MLE-12 cells. cDNA synthesis of miRNA was performed by miRNA reverse transcription kit (TaKaRa). PrimeScript™ RT Reagent kit (TaKaRa, Japan) was taken to produce cDNA for mRNA and circRNA. Next, PCR was completed for each sample in three replicates using the SYBR Green PCR Premix Kit (Invitrogen) on a CFX96 Contact Real-Time Fluorescent Quantitative PCR Detection System (Bio-Rad, CA, USA). The 2^−ΔΔCt^ method was applied to calculate RNA expression levels, which were standardized by GAPDH or U6. Table 1 presents the primer sequences.

**Table 1:** Primer sequence.

**Gene**	**Forward primer (5′ →3′)**	**Reverse primer (5′ →3′)**
circRBM33	5′-GTATGAAGGCCACGAAGCTG-3′	5′-AGCTCTTCTTCTCCACGACC-3′
EZH1	5′-GCTTCCTTCACCCTTTTCATGCCACCC-3′	5′-CGACGACCAGAGCACTTGGAG-3′
miR-15a-5p	5′-GCCGAGTAGCAGCACATAATGG-3′	5′-GTCGTATCCAGTGCAGGGTCCGAGGTATTCGCACTGGATACGACCACAAA-3′
GAPDH	5′-AGCCCAAGATGCCCTTCAGT-3′	5′-CCGTGTTCCTACCCCCAATG-3ʹ
U6	5′-CTCGCTTCGGCAGCACA-3′	5′-AACGCTTCACGAATTTGCGT-3

### Western blot

The MLE-12 cells and tissues underwent two washes with chilled PBS and were then lysed on ice for 20 min using RIPA lysis buffer (FD008, Vazyme). The Pierce ALI Protein Assay Kit (Rockford) was utilized to measure protein levels. Proteins were separated using 10% SDS-PAGE and transferred to PVDF membranes (Millipore). They were closed with 5% skim milk for 2 h and mixed overnight at 4 °C with primary antibodies EZH1 (ab263961, Abcam), GAPDH (Abcam; ab37168), Bax (Abcam; ab32503), Bcl-2 (Abcam; ab182858), Nuclear factor erythroid 2-related factor 2 (Nrf2; Abcam; ab62352), p65 (Abcam; ab32536), and p-p65 (Abcam; ab76302) and with the secondary antibody (Abcam; ab205719) for 1 h at 37 °C. Finally, the results were visualized with the ECL Detection Kit (E411–04, Vazyme) and checked on the FluorChem™M system.

### Actinomycin D assay

Actinomycin D (Sigma, Germany) was added at 5 µg/ml to the whole medium of MLE-12 cells. At the indicated time points, total RNA was extracted from MLE-12 cells, and RT-qPCR was carried out to study circRBM33 and linear RBM33.

### RNase R treatment

RNA was digested with 3U/μg RNase R reagent (Epicentre, WI, USA) at 37 °C for 15 min, and RNA purification was conducted with the RNeasy MinElute Cleanup kit (Qiagen, Hilden, Germany). Subsequently, RT-qPCR was conducted to study circRBM33 expression and linear RBM33.

### Cell viability assay

MLE-12 cells underwent inoculation in 96-well plates at 5 × 10^3^ and were incubated for 12 h Following a 24-hour treatment period, MTT solution (5 mg/ml, 20 μl/well) was introduced for 4 h at 37 °C. Dissolving the insoluble metazoan crystals in 150 μl of dimethyl sulfoxide (Sigma-Aldrich, USA) for 10 min, the absorbance at 490 nm was observed using a microplate reader (Sanco, China).

### Flow cytometry

By using the Annexin V-FITC/PI Apoptosis Detection Kit (Invitrogen), the apoptosis rate was determined. MLE-12 cells, totaling 1 × 10^5^ cells, underwent resuspension and were cleansed twice using pre-cooled PBS. The cells underwent resuspension in 500 μl 1 × Binding Buffer, followed by 5 μl Annexin V-FITC and propidium iodide, respectively. Subsequently, the proportion of apoptotic cells was examined using a FACScan® flow cytometer (BD Biosciences, USA) post a 30-min staining period.

### Dual luciferase assay

Potential binding sites for miR-15a-5p and circRBM33 or EZH1 were predicted in the starBase 3.0 (http://starbase.sysu.edu.cn/). Wild-type (wild-type) or mutant (mutant) circRBM33 fragment and EZH1 containing miR-15a-5p binding site were synthesized by GenePharma (Shanghai, China), wild-type reporter vectors (circRBM33-WT and EZH1-WT) and mutant reporter vectors (circRBM33-MUT and EZH1-MUT) were produced by cloning the fragments into pmirGLO vectors (Promega, WI, USA). The luciferase reporter plasmid was co-transfected into MLE-12 cells (1 × 10^4^ cells/well on 96-well plates) with miR-15a-5p mimic or mimic-NC using Lipofectamine®3000 (Invitrogen) into MLE-12 cells, and the culture was continued for 48 h The luciferase reporter plasmid was assayed by the Dual Luciferase Assay System (Promega).

### RNA immunoprecipitation (RIP)

RIP assays were conducted using the Magna RIP Kit (Millipore, MA, USA). MLE-12 cells were lysed with RIP lysis buffer, and 10 μL cell lysates were co-incubated with magnetic beads containing anti-Ago2 (AALIm, MA, USA) or anti-IgG (AALIm) at 4 °C for 6 h Immunoprecipitates bound to magnetic beads were eluted, and the enriched levels of circRBM33, miR-15a-5p, and EZH1 were analyzed by RT-qPCR after RNA purification.

### Determination of oxidative stress

Newly prepared lung homogenates or cells underwent incubation with 2′,7′-Dichlorodihydrofluorescein diacetate (DCFH-DA; 50 μmol/L) for half an hour at 37 °C in darkness, with the outcomes being monitored through a fluorescence microscope (Leica). Malondialdehyde (MDA) content, Superoxide Dismutase (SOD) activity, and glutathione (GSH) were measured by commercial Enzyme-linked immunosorbent assay (ELISA) kits (Abcam, Cambridge, MA, USA).

### ELISA assay

Tumor necrosis factor-α (TNF-α) and interleukin-6(IL-6) concentrations in lung tissues and MLE-12 cells were determined using ELISA Kits (R&D Systems, MN, USA).

### Establishment of septic ALI mouse model

Adult male C57BL/6 mice, ranging in age from 8 to 9 weeks and sourced from Beijing Vital River Laboratory Animal Technology Co., Ltd. in Beijing, China, resided in a regulated setting with temperatures between 22–24 °C and 60% humidity, experiencing a 24-hour light/dark rhythm. Availability of food and water was ensured. All animal studies followed the ARRIVE guidelines. Following a one-week acclimatization period, cecum ligation and puncture (CLP) were performed using a 22-gauge needle to establish the mouse model. Prior to the procedure, the mice were anesthetized with 40 mg/kg sodium pentobarbital (2% (w/v)). The removal of hair from the abdomen was followed by a disinfection of the abdominal skin using iodine. Following ejection of some feces from the perforation, the tied cecum was reinserted into the peritoneal cavity, and the abdominal cut was carefully closed. Post-surgery, mice received subcutaneous injections of saline at six-hour intervals to ensure fluid resuscitation. The completion of CLP surgery in each mouse occurred within a span of ten minutes. In the sham-operated group, mice underwent the same surgical procedure as in open surgery, with the exception of the CLP surgery.

To generate mice with downregulation of circRBM33 in lung tissues, an intravenous injection of short hairpin RNA specific to the circRBM33 (AAV8-shRNA-circRBM33; Shandong WZ Biotechnology Co., Ltd.) (20 μL, 1 × 10^7^ vg/mL) or AAV8-shRNA-scramble (AAV8-shRNA-sc; Hanheng Biotechnology (Shanghai) Co., Ltd.) was performed 7 days before CLP surgery.

After 6 weeks, mice were euthanized by sodium pentobarbital at 250 mg/kg by intraperitoneal injection, and mouse lung tissue was obtained and stored in 4% paraformaldehyde (Beyotime). Mouse eyelids were collected and sodium citrate 4% (Beyotime) was added to the blood collected. Serum was obtained by centrifugation of the plasma at 4000 × g for 10 min at 4 °C.

### Wet and dry weight (W/D) ratio

The middle lobe of the right lung weighed (W) after surface moisture was absorbed. The tissue was then dehydrated at 80 °C for 48 h to obtain the dry lung weight (D). Lung edema index = W/D.

### HE staining and histologic damage scoring

The procedure involved immersing lung tissues in 4% paraformaldehyde for a day, followed by dehydration and embedding in paraffin. They were then sliced coronally to a 5 μm thickness, dyed using hematoxylin-eosin (Beyotime), treated with 1% hydrochloric acid in alcohol for 10 minutes, washed with 2% sodium bicarbonate (Beyotime) for 10 s, and finally stained with eosin for an additional 3 min. After dehydration of the sections with graded alcohol, they were washed with xylene and sealed with neutral resin. Using an Olympus BX 53 microscope (Tokyo, Japan). The Lung Injury Score (including hemorrhage, inflammation, and edema) was graded on the following scale: 0 being normal, 2 being mild, 4 being moderate, 6 being severe, and 8 being extremely severe.

### Immunohistochemistry

Samples of lung tissue were preserved in 4% paraformaldehyde, dehydrated using ethanol, and encased in paraffin. Slices of the tissues were cut down to a thickness of 4 μm and then arranged on slides. Post-deparaffinization using xylene (Beyotime), the tissue samples underwent a PBS wash, were treated with 0.3% H_2_O_2_ for 10 min at ambient temperature, non-specifically bonded with PBS infused with 5% FBS and 0.3% Triton X-100 for an hour, followed by an overnight incubation with Bax and Nrf2 primary antibodies (Abcam) at 4 °C. After secondary antibody detection (ab6720, Abcam) for 1 h at ambient temperature, the target signals were developed by DAB substrate (Vector Labs, CA, USA) and the slides were re-stained with hematoxylin for 2 min, and finally visualized using microscopy (Leica).

### Immunofluorescence

The sections of tissue underwent a rinse with PBS, were stabilized using 4% paraformaldehyde, and made permeable using 0.5% Triton X-100. Post a 30-min sealing using 5% BSA, the samples underwent an overnight detection at 4 °C with Bax and Nrf2 primary antibody (Abcam), succeeded by a 30-min detection at 37 °C with Alexa Fluor 488- and Alexa Fluor 555-labeled IgG. Nuclei underwent staining using DAPI, and the outcomes were assessed under a light microscope.

### Data analysis

The experimental data were statistically analyzed using SPSS20 statistical software. For comparing two groups, *t*-tests were used, and for comparing multiple groups, one-way variances were tested. Statistics were considered significant at *P* < 0.05.

## Results

### circRBM33 is elevated in ALI

**Fig. 1:**
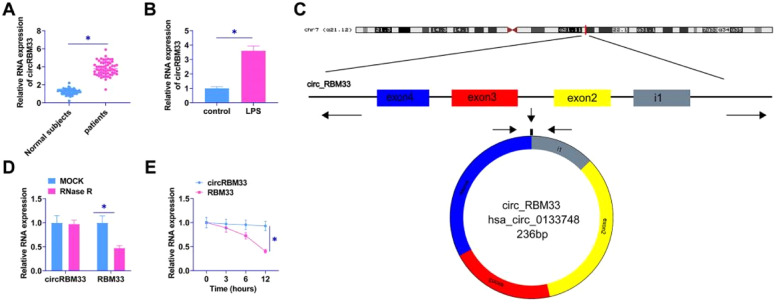
circRBM33 is highly expressed in ALI. A: RT-qPCR for circRBM33 expression in ALI tissues; B: RT-qPCR for circRBM33 expression in MLE-12 cells; C: circRBM33 structure; D: Validation of circRBM33 stability by RNase R treatment and RT-qPCR analysis; E: RT-qPCR detection of Actinomycin D-treated circRBM33 content in different time points. Data are expressed as mean ± SD (n = 3). * *P* < 0.05.

The authors examined circRBM33 in the plasma of sepsis patients and MLE-12 cells by RT-qPCR. circRBM33 was upregulated in the plasma of sepsis patients (Fig. 1A), and high levels of circRBM33 were also shown in MLE-12 cells (Fig. 1B). Using Bioinformatics Degree Points (www.circbase.org/) and Primer, the authors determined the circular structure of circRBM33 transcripts (Fig. 1C). A RNase R and actinomycin D assay was performed next. Results indicated that circRBM33 was resistant to RNase R, but NMNAT1 was rapidly digested (Fig. 1D). A comparison of circRBM33 and RBM33 showed that circRBM33 was more stable than RBM33 under actinomycin D (Fig. 1E).

### circRBM33 deficiency delays LPS-induced damage in MLE-12 cells

**Fig. 2:**
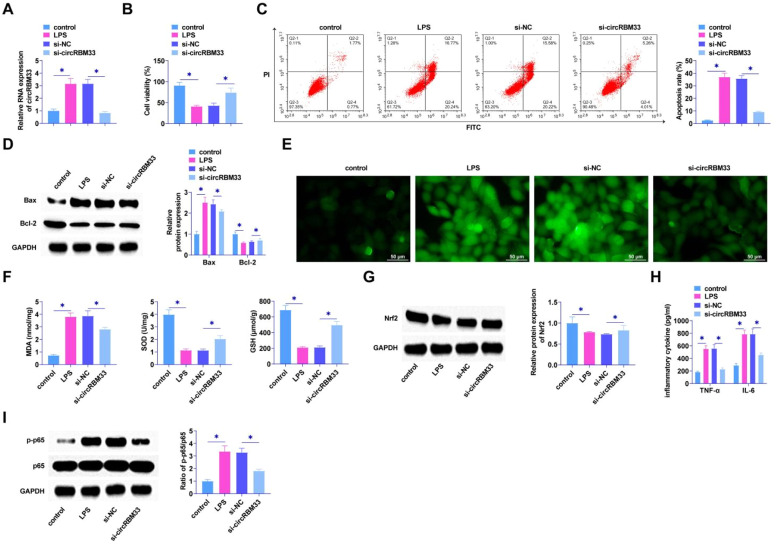
Knockdown of circRBM33 delays MLE-12 cell injury. Si-circRBM33 or si-NC was transfected into MLE-12 cells. A, RT-qPCR for circRBM33; B, MTT assay to determine the viability of MLE-12 cells; C, Flow cytometry to determine apoptosis rate of MLE-12 cells; D, Western Blot to determine the expression levels of Bax and Bcl-2; E, ROS activity in MLE-12 cells; F, Kit assay for MDA, SOD and GSH content in MLE-12 cells; G, Western Blot assay for protein expression of Nrf2; H, ELISA assay for TNF-α, IL-6 levels; I, Western Blot assay for p-p65 expression. Data are expressed as mean ± SD (n = 3). * *P* < 0.01.

Loss-of-function assay was determined using siRNA targeting circRBM33 in MLE-12 cells. RT-qPCR observed circRBM33 low expression in MLE-12 cells after si-circRBM33 intervention (Fig. 2A), indicating successful transfection. The results by MTT assay presented that the viability of MLE-12 cells was restored after knockdown of circRBM33 (Fig. 2B). In addition, flow cytometry manifested that down-regulating circRBM33 significantly suppressed MLE-12 cell apoptosis rate (Fig. 2C). Western Blot assay demonstrated that down-regulating circRBM33 inhibited Bax expression, while elevated Bcl-2 expression in MLE-12 cells (Fig. 2D). LPS injection significantly increased ROS production, which was inhibited by downregulation of circRBM33 (Fig. 2E). The level of lipid peroxidation product MDA was also reduced in si-circRBM33-treated MLE-12 cells. In addition, down-regulating circRBM33 also significantly inhibited LPS-induced SOD and GSH depletion (Fig. 2F). Western Blot assay showed that down-regulating circRBM33 effectively enhanced Nrf2 in LPS-induced MLE-12 cells (Fig. 2G). Silencing circRBM33 was shown by ELISA assay to reduce TNF-α and IL-6 (Fig. 2H). When inflammation occurs, the transcription factor NF-B plays a central role [38, 39], and the Western Blot results showed that silencing circRBM33 inhibited LPS-induced p65 phosphorylation in MLE-12 cells (Fig. 2I).

### circRBM33 competitively binds miR-15a-5p

**Fig. 3:**
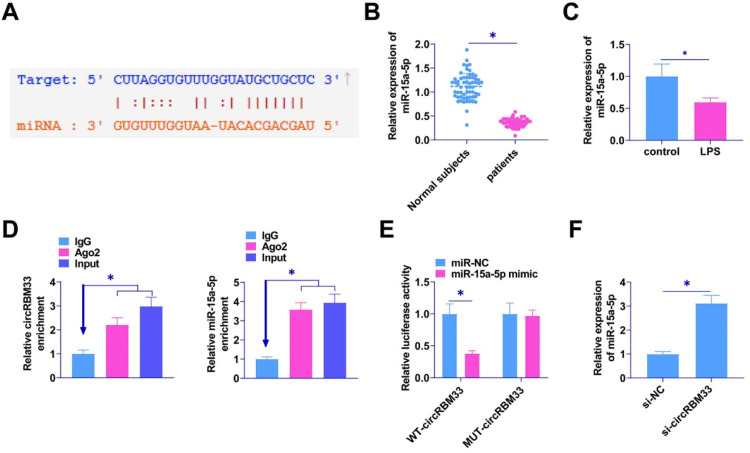
circRBM33 competitively binds miR-15a-5p. A, Starbase predicted the binding site of miR-15a-5p to circRBM33; B, RT-qPCR for miR-15a-5p in plasma of sepsis patients; C, RT-qPCR for miR-15a-5p in MLE-12 cells; D, RIP assay to detect enrichment of circRBM33 with miR-15a-5p in Ago2; E, Dual luciferase reporter assay to detect the direct targeting relationship between miR-15a-5p and circRBM33; F, RT-qPCR assay to show the effect of knockdown of circRBM33 on miR-15a-5p expression. Data are expressed as mean ± SD (n = 3). * *P* < 0.01.

Potential target miRNAs of circRBM33 were searched by the bioinformatics website starBase 3.0. The results confirmed the existence of a potential binding site for miR-15a-5p and circRBM33 (Fig. 3A). RT-qPCR was conducted to detect miR-15a-5p in the plasma of sepsis patients and in the MLE-12 cells. miR-15a-5p in the plasma of sepsis patients was lower than that in plasma of normal subjects (Fig. 3B), and the expression level in MLE-12 cells induced by LPS was downregulated (Fig. 3C). RIP assay experiments manifested that both circRBM33 and miR-15a-5p were highly enriched via Ago2 incubation (Fig. 3D). MiR-15a-5p mimic co-transfected with WT-circRBM33 decreased luciferase activity in dual-luciferase reporter assays (Fig. 3E). RT-qPCR assay found that knocking down circRBM33 enhanced miR-15a-5p expression (Fig. 3F).

### CircRBM33 targeting miR-15a-5p affects ALI development

**Fig. 4:**
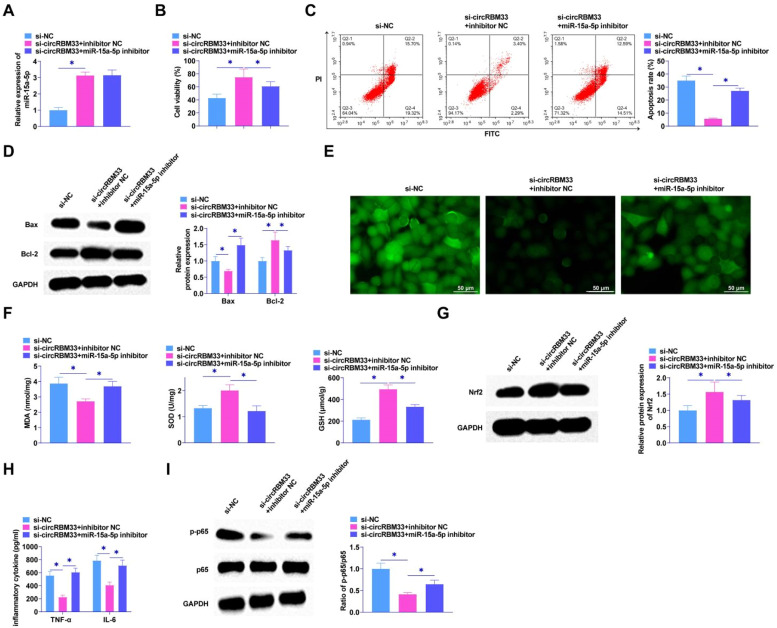
circRBM33 targeting miR-15a-5p affects ALI development. Si-circRBM33 and miR-15a-5p inhibitor were co-transfected into MLE-12 cells. A, RT-qPCR to determine miR-654–3p after co-transfection; B, MTT to detect the proliferation of MLE-12 cells; C, Flow cytometry to detect apoptosis; D, Western Blot to determine Bax and Bcl-2 expression levels; E, ROS activity in MLE-12 cells; F, Kit assay for MDA, SOD and GSH content in MLE-12 cells; G, Western Blot assay for protein expression of Nrf2; H, ELISA assay for TNF-α and IL-6 levels; I, Western Blot assay for p-p65 expression. Data are expressed as mean ± SD (n = 3). * *P* < 0.05.

The interrelationship between circRBM33 and miR-15a-5p in MLE-12 cells was explored by functional rescue experiments to elucidate whether miR-15a-5p exerts an effect in circRBM33 mediation in MLE-12 cells. Co-transfection of si-circRBM33 and miR-15a-5p inhibitor into MLE-12 cells using RT-qPCR assay confirmed that knockdown of circRBM33 augmented miR-15a-5p expression (Fig. 4A). MTT assay revealed that miR-15a-5p inhibitor attenuated the influences of si-circRBM33 on MLE-12 cell viability (Fig. 4B). Flow cytometric analysis showed circRBM33 knockdown inhibited apoptosis of MLE-12 cells, but miR-15a-5p inhibitor co-transfected cells showed increased apoptosis (Fig. 4C). As determined by Western Blot, miR-15a-5p downregulation reduced circRBM33 silencing's effect on Bax and Bcl-2 expression (Fig. 4D). As found in Fig. 4E, miR-15a-5p inhibition prevented the function of circRBM33 downregulation on ROS production. Down-regulating circRBM33 also significantly reduced MDA levels, but miR-15a-5p inhibition impeded the effect. In addition, miR-15a-5p inhibition impaired the promotion of SOD and GSH production mediated by down-regulation of circRBM33 (Fig. 4F). Western blot assayed that down-regulating circRBM33 effectively enhanced Nrf2 in LPS-stimulated MLE-12 cells, while miR-15a-5p inhibition saved this effect (Fig. 4G). miR-15a-5p down-regulation enhanced TNF-α and IL-6 by ELISA assay, reversing the effect of circRBM33 silencing (Fig. 4H). Western Blot results analyzed that silencing of circRBM33 inhibited the LPS-induced phosphorylation of p65 in MLE-12 cells, but p65 phosphorylation was enhanced by inhibiting miR-15a-5p (Fig. 4I).

### EZH1 is mediated by miR-15a-5p

**Fig. 5:**
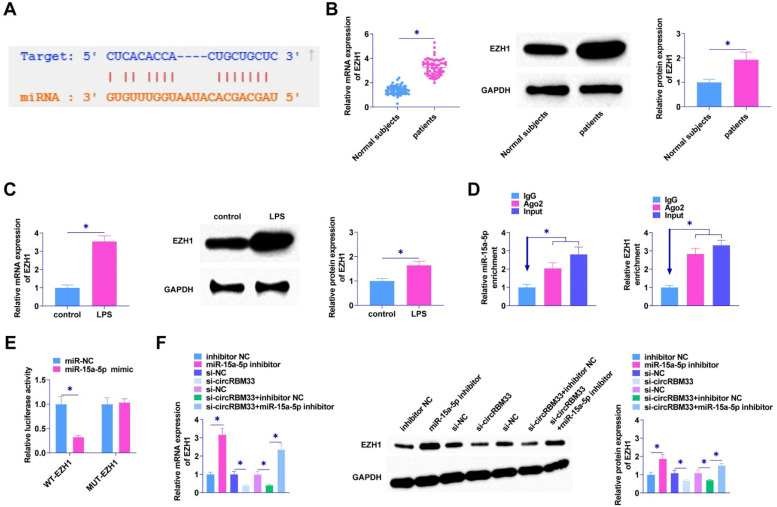
EZH1 is the target gene of miR-15a-5p. A, starBase 3.0 predicted the binding site of miR-15a-5p and EZH1; B, RT-qPCR and Western Blot to detect EZH1 in the plasma of sepsis patients; C, RT-qPCR and Western Blot to detect EZH1 in MLE-12 cells; D, RIP assay to determine miR-15a-5p binding to EZH1; E, Dual luciferase reporter assay to detect the targeting relationship between miR-15a-5p and EZH1; F, RT-qPCR and Western Blot to detect EZH1 in MLE-12 cells. Data are expressed as mean ± SD (n = 3). * *P* < 0.05.

As predicted by the bioinformatics website starBase 3.0, miR-15a-5p targets EZH1 which has a binding site for miR-15a-5p (Fig. 5A), and EZH1 in ALI was determined by RT-qPCR and Western Blot, and it was found in the plasma of patients with sepsis and in MLE-12 cells with a high expression of EZH1 (Fig. 5B and C). EZH1 and miR-15a-5p were shown to be enriched in Ago2 immunomagnetic beads compared to IgG immunoprecipitation by RIP assay (Fig. 5D). The dual luciferase reporter gene assay verified the interaction area between EZH1 and miR-15a-5p, revealing a notable reduction in luciferase activity following the co-transfection of miR-15a-5p and WT-EZH1 (Fig. 5E). miR-15a-5p inhibitor intervention promoted EZH1 levels in MLE-12 cells. si-circRBM33 was first transfected into MLE-12 cells, and down-regulating circRBM33 inhibited EZH1 expression in MLE-12 cells. However, down-regulating miR-15a-5p suppressed the effect of circRBM33 on EZH1 expression (Fig. 5F).

### EZH1 overexpression hampers the effect of circRBM33 knockdown on ALI development

**Fig. 6:**
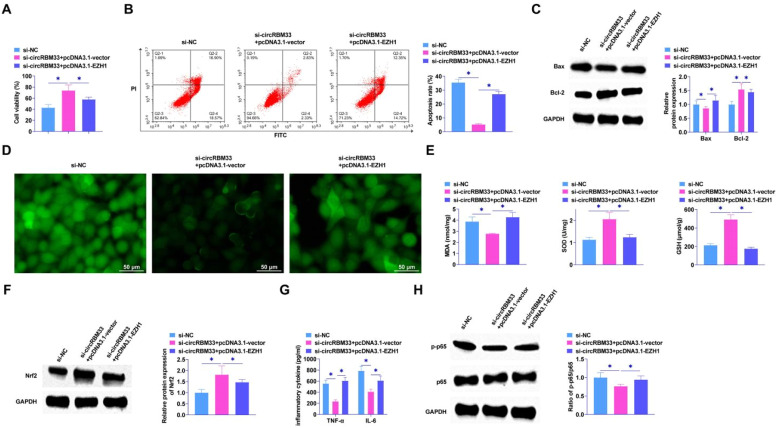
Overexpression of EZH1 reverses the inhibitory effect of circRBM33 knockdown on ALI development. si-circRBM33 and pcDNA 3.1-EZH1 were co-transfected into MLE-12 cells. A, MTT assay for MLE-12 cell proliferation; B, Flow cytometry for apoptosis; C, Western Blot for Bax and Bcl-2 expression levels; D, ROS activity in MLE-12 cells; E, Kit assay for MDA, SOD and GSH content; F, Western Blot for protein expression of Nrf2; G, ELISA for TNF-α and IL-6 levels; H: Western Blot for p-p65 expression. Data are expressed as mean ± SD (n = 3). * *P* < 0.01.

The knockdown of circRBM33 was further confirmed by functional rescue assay to inhibit the progression of ALI through the miR-15a-5p/EZH1 axis. After co-transfection of pcDNA3.1-EZH1 and si-circRBM33 into MLE-12 cells, MTT assay showed that EZH1 overexpression saved the effect of si-circRBM33 on MLE-12 cell viability (Fig. 6A). Reducing circRBM33 inhibited apoptosis of MLE-12 cells, but overexpression of EZH1 increased MLE-12 cell apoptosis rate (Fig. 6B). Western Blot also showed the same result that overexpression of EZH1 mitigated the effect of silencing circRBM33 on Bax and Bcl-2 (Fig. 6C). As shown in Fig. 6D, circRBM33 knockdown inhibited ROS production and overexpression of EZH1 reversed the effect. Also overexpressing EZH1 reversed the inhibition of MDA and the promotion of SOD and GSH by downregulation of circRBM33 (Fig. 6E). Western blot assay showed that overexpressing EZH1 disrupted the promotion of Nrf2 protein expression by down-regulated circRBM33 (Fig. 6F). EZH1 overexpression was shown to reverse the effect of si-circRBM33 on cellular inflammatory response and increase TNF-α and IL-6 as determined by ELISA (Fig. 6G). Western Blot results showed that EZH1 overexpression prevented the effect of silencing circRBM33 on p65 phosphorylation (Fig. 6H).

### Suppressing circRBM33 inhibits ALI *in vivo*

**Fig. 7:**
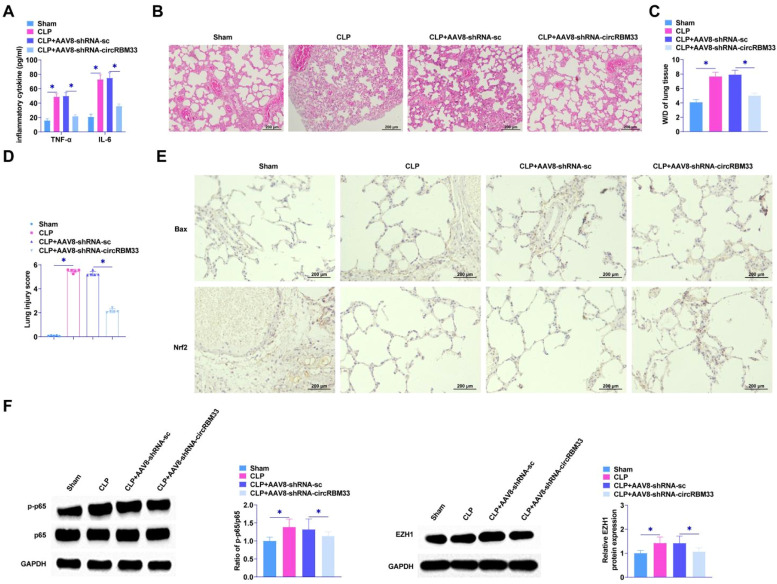
circRBM33 down-regulation inhibits ALI *in vivo*. A, ELISA to determine TNF-α and IL-6 in mouse lung tissues; B, HE staining to assess lung tissue injury; C, W/D of lung tissues in mice after CLP surgery; D, Intensity of injury scoring; E, Immunohistochemistry to detect Bax and Nrf2; F: Western Blot to analyze p-p65 and EZH1 in ALI mice. Data are expressed as mean ± SD (n = 5). * *P* < 0.05.

The role of circRBM33 in ALI was further assessed by injecting AAV8-shRNA-circRBM33 or AAV8-shRNA-sc into mice. To determine the effect of circRBM33 on sepsis severity, the authors first measured the levels of proinflammatory factors by ELISA, which are biomarkers of injury and inflammation in sepsis and sepsis-associated ALI. The results determined that down-regulating circRBM33 decreased TNF-α and IL-6 and inhibited pro-inflammatory factor release in lung tissues (Fig. 7A). Histological analysis showed that the sham-operated group exhibited alveoli with normal morphology. In contrast, the CLP group exhibited alveolar sac collapse, as well as thickening of alveolar walls and septa. In addition, vascular congestion and hemorrhage were seen in the CLP group, and down-regulating circRBM33 ameliorated lung tissue injury (Fig. 7B). Pulmonary edema was analyzed using the W/D test. CLP surgery enhanced W/D in mice, whereas downregulation of circRBM33 attenuated this effect (Fig. 7C). In addition, the injury intensity score was higher in the CLP group than in the sham-operated group, whereas the injury score was lower in the CLP + AAV8-shRNA-circRBM33 group than in the CLP group (Fig. 7D). Immunohistochemistry results showed that down-regulating circRBM33 suppressed the apoptosis level of lung tissue cells and decreased Bax, while promoting Nrf2 (Fig. 7E). Meanwhile, down-regulating circRBM33 inhibited the phosphorylation of p65 in ALI and decreased EZH1 in septic ALI (Fig. 7F).

## Discussion

In sepsis, multiple organs are affected, primarily the lungs and kidneys, due to a systemic inflammatory response. To date, treatment of sepsis has some limitations, and morbidity and mortality rates have not been reduced significantly. A new therapeutic strategy for sepsis, based on early diagnosis, is necessary. CircRNA is an endogenous RNA that is involved in many diseases as a regulator of gene expression. In addition, circRNAs can be used as potential biomarkers for diseases and disorders. It has been shown that septic ALI is associated with apoptosis and the production of inflammatory factors in lung cells,^20^ processes that are regulated through circRNAs.

In MLE-12 cells, circRBM33 was investigated for its effects on LPS-induced apoptosis and inflammation and CLP-induced ALI. CLP surgery is a commonly employed *in vivo* sepsis induction model, as its pathophysiology is similar to human sepsis. A mouse model of CLP-induced ALI was successfully established in the present study. Lung tissue in the CLP group showed severe morphological changes, including alveolar thickening, hemorrhage, and alveolar collapse. Increased proinflammatory mediators and apoptosis play an important role in the development of sepsis-induced ALI. NF-κB is a transcription factor critical for inflammatory responses, and its subunit p65 is acetylated at multiple lysine sites, facilitating the binding of NF-κB to its downstream proinflammatory factors.^21^ LPS is a lipid endotoxin of Gram-negative bacteria and is often used to induce ALI in experiments. Both CLP group and LPS-treated MEL-12 showed a significant increase in the level of inflammatory cytokines and activation of NF-κB. Bax and Bcl-2 are important proteins associated with apoptosis, and the expression of Bax was elevated in septic patients and mice with ALI, whereas the expression of Bcl-2 was decreased. The same results were shown in LPS-induced MLE-12 cells. circRBM33 silencing restored CLP- and LPS-induced lesion, inflammation, and apoptosis and attenuated ALI progression. To the best of our knowledge, this report is the first study proposed to explore the mechanism of circRBM33′s role in sepsis-induced ALI, providing new molecular targets for the prediction of sepsis-induced ALI.

Oxidative stress is a key process that promotes sepsis and can trigger impaired vascular permeability mitochondrial dysfunction, ultimately leading to multi-organ dysfunction and death.^22^ LPS triggered the accumulation of ROS and MDA and inhibited SOD and GSH activities, but the silencing of circRBM33 reversed the performance of LPS. Nrf2 is a modifier of antioxidant and detoxifying cellular responses and was highly expressed in sepsis. Nrf2 regulates the innate immune response during sepsis and improves survival by regulating GSH and other antioxidant enzymes to maintain redox homeostasis and inhibit dysregulation of pro-inflammatory signaling pathways.^23^^,^^24^ The mechanisms of Nrf2 activation and protection remain to be determined.

Sepsis is strongly influenced by miRNAs.^25^^,^^26^ A previous study has shown that pro-inflammatory factors are enhanced in LPS-treated RAW264.7 macrophages and that inhibition of miR-15a-5p expression blocks inflammation and acts as an anti-inflammatory in sepsis.^27^ In contrast, the present experimental results showed that miR-15a-5p was significantly downregulated in sepsis-induced ALI. Xu et al. established a miRNA microarray dataset and identified miRNAs that were differentially expressed in sepsis-induced acute kidney injury, of which miR-15a-5p was downregulated.^28^ The results are in line with ours. This suggests that miR-15a-5p has different expression patterns in different diseases. In addition, miR-15a-5p plays an important role in the process of lung injury. For example, miR-15a-5p regulates acute lung injury due to traumatic hemorrhagic shock.^16^ PRKCA promotes mitochondrial autophagy via the miR-15a-5p/PDK4 axis to alleviate sepsis-induced ALI.^29^ The main feature of ALI is that it causes edema and massive infiltration of inflammatory cells in the lung tissue, which further develops into irreversible lung tissue damage.^30^ In this study, down-regulating miR-15a-5p accelerated the emergence of apoptosis and inflammation in LPS-treated MLE-12 cells and rescued the suppressive effect of silencing circRBM33 on septic ALI.

MiRNAs mediate gene expression by binding to target sequences.^31^ MiR-15a-5p negatively regulated EZH1 expression, as confirmed. It was shown by rescue experiments that EZH1 overexpression reduced the ameliorative effects of silencing circRBM33 on apoptosis and inflammatory responses in LPS-treated MLE-12 cells. Meanwhile, the summarized results suggest that the circRBM33/miR-15a-5p/EZH1 axis can be used as a clinical diagnostic marker of the disease and a tool for the search for targeted drugs, which is expected to solve the challenge of the lack of specificity of the currently commonly used clinical biomarkers.

With further research, the different expression of circRNAs in cells is an important factor in sepsis formation and evolution.^32^ Damage to the ceRNA network axis affects sepsis-associated cell proliferation, death, and self-consumption and triggers the formation of inflammatory factors.^33^ Thus ceRNA may become an early diagnostic indicator and therapeutic target for sepsis. However, studies on the regulatory network of ceRNAs in sepsis-induced ALI are still in the preliminary stage, and further refinement and deepening of the complex ceRNA network is needed. Compared with other circRNA/miRNA/mRNA axis studies of sepsis-induced ALI, the present study breaks through the previous limitation of analyzing the ceRNA regulatory network based on bioinformatics only and combines *in vitro* and *in vivo* experiments for further validation. This can provide a more in-depth understanding of the pathogenesis of sepsis mediated by ceRNAs, and can also lay the foundation for the development of drugs and intervention targets for sepsis patients.

However, this study only initially explored the possible protective mechanism of circRBM33 against sepsis-induced ALI, and its upstream and/or downstream signaling molecule regulatory network and its exact mechanism have not been elucidated; therefore, the next step will be to use transgenic mice and multi-omics analysis to gain a more comprehensive understanding of the exact protective mechanism of circRBM33 in this disease. The sample size collected in the clinical trial of this study was insufficient, which may cause some errors. Later, the authors will increase the sample size to further investigate the exact mechanism of circRBM33/miR-15a-5p/EZH1 axis in sepsis-induced ALI. In this study, only pro-inflammatory factors were detected in ALI, but both pro-inflammatory and anti-inflammatory factors can be produced during the development of ALI. The authors will add the detection of anti-inflammatory factors later to reflect the development of inflammatory response in ALI more comprehensively.

In summary, the present study found that the knockdown of circRBM33 could exert a protective effect against sepsis-induced ALI by inhibiting oxidative stress, inflammatory response and apoptosis. The above results provide a new theoretical basis for circRBM33 to be a potential clinical therapeutic target for sepsis-induced ALI.

## Ethics approval

The present study was approved by the Ethics Committee of The First Affiliated Hospital of Fujian Medical University and written informed consent was provided by all patients prior to the study start. All procedures were performed in accordance with the ethical standards of the Institutional Review Board and The Declaration of Helsinki, and its later amendments or comparable ethical standards (No. 201903FJ13).

The present study was approved by the Animal experiments were approved by The First Affiliated Hospital of Fujian Medical University Animal Experimental Ethics Committee. And all procedures complied with the National Institutes of Health Guide for the Use of Laboratory Animals (No. 20201F631).

## Authors' contributions

Jinquan Lin, Qiongying Wei and Zhipeng Fang designed the research study. Qiongying Wei and Zhipeng Fang performed the research. Jinquan Lin and Qiongying Wei provided help and advice on the experiments. Jinquan Lin, Qiongying Wei and Zhipeng Fang analyzed the data. Jinquan Lin, Qiongying Wei and Zhipeng Fang wrote the manuscript. Jinquan Lin and Qiongying Wei reviewed and edited the manuscript. All authors contributed to editorial changes in the manuscript. All authors read and approved the final manuscript.

## Declaration of competing interest

The authors declare no conflicts of interest.

## Data Availability

The datasets used and/or analyzed during the present study are available from the corresponding author upon reasonable request.
